# Analysis of Methylglyoxal Concentration in a Group of Patients with Newly Diagnosed Prediabetes

**DOI:** 10.3390/biomedicines11112968

**Published:** 2023-11-03

**Authors:** Edyta Sutkowska, Izabela Fecka, Dominik Marciniak, Katarzyna Bednarska, Magdalena Sutkowska, Katarzyna Hap

**Affiliations:** 1 University Rehabilitation Centre, Wroclaw Medical University, Borowska 213, 50-556 Wroclaw, Poland; katarzyna.hap@umw.edu.pl; 2 Department of Pharmacognosy, Faculty of Pharmacy, Wroclaw Medical University, Borowska 211, 50-556 Wroclaw, Poland; izabela.fecka@umw.edu.pl (I.F.); katarzyna.bednarska@student.umed.wroc.pl (K.B.); 3 The Committee on Therapeutics and Pharmaceutical Sciences, The Polish Academy of Sciences, pl. Defilad 1, 00-901 Warszawa, Poland; 4 Department of Drugs Form Technology, Wroclaw Medical University, Borowska 211, 50-556 Wroclaw, Poland; dominik.marciniak@umw.edu.pl; 5 Faculty of Medicine, Wroclaw Medical University, Wybrzeże Ludwika Pasteura 1, 50-367 Wroclaw, Poland; m.sutkowska@student.umw.edu.pl

**Keywords:** prediabetes, methylglyoxal, advanced glycation end-products, diabetic chronic complications

## Abstract

Background: The abnormal serum concentration of methylglyoxal (MGO) has been presented as an indicator of chronic complications in diabetes (DM). Because such complications are also found in pre-DM, we decided to assess the concentration of this compound in individuals with pre-DM, without cardio-vascular diseases. Methods: Frozen samples from individuals newly diagnosed with pre-DM (N = 31) and healthy subjects (N = 11) were prepared and MGO concentration was determined using UHPLC-ESI-QqTOF-MS. Results: Statistical significance was established when the groups were compared for body weight, BMI, fasting glucose level, fatty liver and use of statins but not for the other descriptive parameters. The positive linear correlation showed that the higher HbA1c, the higher MGO concentration (*p* = 0.01). The values of MGO were within the normal range in both groups (mean value for pre-DM: 135.44 nM (±SD = 32.67) and for the control group: 143.25 nM (±SD = 17.93); *p* = 0.46 (±95% CI)), with no statistical significance between the groups. Conclusions: We did not confirm the elevated MGO levels in the group of patients with pre-DM. The available data suggests a possible effect of statin intake on MGO levels. This thesis requires confirmation on a larger number of patients with an assessment of MGO levels before and after the introduction of statins.

## 1. Introduction

Prediabetes (pre-DM) can lead to diabetes mellitus (DM) [1] and because, in most cases, there is a conglomeration of numerous cardiovascular risk factors (the so-called metabolic syndrome) [2], it remains in the attention of the medical community in the context of treatment and prevention [3,4,5]. There are two types of pre-DM [6]: impaired fasting glucose (IFG) and impaired glucose tolerance (IGT) which can exist separately or together (if the patient reaches the criteria for both). Many complications, referred to as chronic diabetic (macro- and microvascular) complications, can be identified among patients with DM. As DM is generally an oligo- or asymptomatic disease (except for most serious cases with very high glucose levels), chronic complications may occur even just after a diagnosis of type 2 diabetes (T2DM). Interestingly, such complications can be found also within the group with pre-DM [7]. The reason for this is still unclear as from the general point of view diabetic complications derive from hyperglycaemia which, however, does not reach sufficient levels in pre-diabetic states. That is why not only glycaemia but also other parameters, which describe metabolic failure both in pre-DM and DM, are taken into account. It is known that even if glycaemia is well-controlled, patients with DM still express a residual risk of chronic complications [7]. This is probably due to risk factor accumulation accompanying carbohydrate disturbances or accumulation of previously introduced harmful functional and cell structure modifications as the effect of long-term metabolic disturbances. One of the main phenomena explaining the formation of those chronic complications is the creation of advanced glycation end-products (AGEs) [8]. Although the most known precursors of AGEs are glucose and fructose [9], an important role in the process is also played by the reactive dicarbonyls [8], with methylglyoxal (MGO) being the most reactive one [10]. The methylglyoxal-derived AGE, responsible for diabetic complications, is called methylglyoxal-derived hydroimidazolone 1 (MG-H1) [11]. The precursors of MGO are the intermediates of glycolysis (glyceraldehyde-3-phosphate and dihydroxyacetonephosphate) but those precursors can also be unrelated to the glucose metabolism pathway and may derive from the metabolism of fatty acid, amino acid, and ascorbic acid [12,13]. Because of those glucose-dependent and glucose-independent formations of AGEs and AGE precursors, with the possible correction of their levels [8] (which is still in the interest of scientific research), information about who could potentially benefit from the treatment is also desirable. It means that first it is required to identify group/s of people with abnormal levels of those substances. The patients at the initial stage of carbohydrate metabolism disorders, such as people with pre-DM, are the first line of research. Confirmation of abnormal levels of the metabolites for AGE formation in this group of patients may explain the potential mechanisms of the occurrence of chronic complications, so far referred to as hyperglycaemic, which possibly are highly glucose-independent.

In The Maastricht Study [14], Hanssen N. and colleagues concluded that MGO levels are associated with microvascular, but not macrovascular disorders. The authors also found higher fasting plasma MGO levels in subjects with prediabetes compared to healthy ones. In the study mentioned, 14.0% of the participants with pre-DM suffered from previously diagnosed cardiovascular disease (CVD). Due to discrepancies regarding the MGO concentration in patients with coronary artery disease in the literature [15,16], we conducted a preliminary analysis only on individuals without a previous history of confirmed coronary artery disease, stroke or intermittent claudication to exclude the potential impact of clinically important ischemia on the results.

The study aimed to assess the MGO concentration in individuals without previous CVD diagnosis who suffer from newly diagnosed prediabetes and to find if the difference between the levels of MGO in healthy individuals and patients with prediabetes exists. The confirmation of a high level of MGO in patients with prediabetes could potentially explain why early complications may start even before the diabetes is diagnosed.

## 2. Materials and Methods

### 2.1. Sample Origin

Frozen serum samples from patients with newly diagnosed IFG and/or IGT diagnosis based on European Association for the Study of Diabetes (EASD) criteria [17], and samples without such pathology were analysed by assessing the concentration of MGO. The material was obtained from Biobank where it had previously been anonymously deposited for another study, after the Bioethics Committee expressed consent for its use (No: KB385/2017).

The serum from both groups was obtained from patients with no previous vascular disease, cancer or any complications referred to as “chronic complications in diabetes” (people with pre-DM). In addition, to characterise the groups and assess the potential impact of other factors on the concentration of MGO, data from the interview, physical examination and the results of basic laboratory tests, which were previously collected in the study in which the subjects participated, were used [18]. The fatty liver was diagnosed only based on the ultrasound examination results obtained from patients’ records.

### 2.2. Sample Preparation and Analysis

The analysis of MGO concentration was carried out according to the method and process suggested in the literature [19,20,21,22], the course of which guarantees the most reliable results.

#### 2.2.1. Chemicals and Standards

Methylglyoxal 40% in water (CAS: 78-98-8), methanol LC-MS grade (CAS: 67-56-1), acetonitrile LC-MS grade (CAS: 75-05-8), water LC-MS grade (CAS: 7732-18-5), formic acid LC-MS grade (CAS: 64-18-6), 4-hydroxyquinazoline (CAS: 491-36-1), perchloric acid 60% (PCA)(CAS: 7601-90-3), 2-methylquinoxaline (CAS: 7251-61-8), o-phenylenediamine (oPD)(CAS: 95-54-5) were all purchased from Merck-Sigma-Aldrich (Poland—Sigma-Aldrich; Poznań, Poland). The stock solution of 4-hydroxyquinazoline used as an internal standard at 6.5 mM was prepared by dissolving the appropriate amount in water. The standard working solution of 2-methylquinoxaline in methanol at 10 mM was used to construct a calibration curve in a range of 10–200 nM.

#### 2.2.2. Sample Preparation for MGO Assay

The sample preparation was performed following a slightly modified method proposed by Scheijen et al. [22]. Before analysis, all plasma samples stored at −80 °C were thawed and mixed thoroughly on a vortex mixer (IKA Poland; Warsaw, Poland). Then, 30 μL of plasma was mixed with 90 μL of o-phenylenediamine (10 mg oPD in 10 mL 1.6 mol/L PCA) in an Eppendorf cup. After 24h of derivatisation at ambient temperature in a dark place, 20 μL of internal standard solution (4-hydroxyquinazoline, 6.5 mM) was added. Samples were again mixed and subsequently centrifuged for 20 min at 15,000× g (MPW MED. Instruments; Warsaw, Poland). The supernatant was transferred into 1.5 mL vials (BIOSENS; Warsaw, Poland) with glass inserts (BIONOVO; Legnica, Poland) and placed in an autosampler (Thermo Fisher Scientific; Waltham, MA, USA). Finally, 5 μL was injected for UHPLC-ESI-QqTOF-MS analysis. All samples were measured in 2 technical replicates. The peak area ratio (analyte/internal standard) was used for the calculation of mean methylglyoxal levels in the samples.

The average value calculated from two measurements for the same sample was used for statistical evaluation.

#### 2.2.3. MGO Determination by UHPLC-ESI-QqTOF-MS

The Thermo Scientific Dionex UltiMate 3000 system (Thermo Fisher Scientific; Waltham, MA, USA) equipped with a quaternary pump (LPG-3400D, Thermo Fisher Scientific; Waltham, MA, USA) and UltiMate 3000RS autosampler (WPS-3000) interfaced with Compact ESI-QqTOF-MS (Bruker Daltonics; Bremen, Germany) was used to determine serum MGO levels in plasma samples. The separation of the MGO derivative was carried out on a Kinetex C18 column (100 mm × 2.1 mm × 2.6 µm) (Phenomenex; Torrance, CA, USA). The analysis was performed according to the same method proposed by Scheijen et al. [22] with slight modifications. Mobile phases consisted of A (0.1% formic acid in water) and B (0.1% acetic acid in 100% acetonitrile). The gradient mobile phase program at a flow rate of 0.4 mL/min was used as follows: 0–4 min, 100–80% A in B; 4–6 min, 80–50% A in B; 6–8 min, 50% A in B; 8–10 min 50–100% A in B. Then, the system returned to the initial setting and was washed with 100% A in B until the system was stabilised before the next analysis. The analysis was carried out in positive ion mode with a scan range of 30–400 m/z. Nitrogen at 2.0 bar pressure, 200 °C temperature, and 0.7 L/min flow was used as the drying and nebuliser gas in the electrospray ionisation interface. The capillary voltage was 4500 V (ESI+). Sodium formate clusters in concentrations of 10 mM were used for internal calibration, with an injection volume of 5 μL. Peaks were analysed using Quant Analysis 2.2 software (Bruker Daltonics; Bremen, Germany).

### 2.3. Statistical Analysis

Statistical analyses were based on a database collected from 42 participants, divided into two independent main groups: a study group (N = 31), and a control group (N = 11).

The variables subjected to statistical analysis were on dichotomous and quotient scales. In the case of variables on quotient scales, their conformity to a normal distribution and homogeneity of variance were determined. The normality of the distribution was verified with the Shapiro–Wilk test, while the Levene and Brown–Forsyth tests were used to verify the homogeneity of variance. All analysed variables in the quotient scales met the normality of the distribution criterion.

To characterise the two groups compared, basic descriptive statistics were determined within each group: size, mean value, standard deviation, 95% significance interval for the mean value (±95% CI) and bifurcation in the case of quotient variables, and count tables with percentages in the case of dichotomous variables.

The statistical significance of the correlations between variables on the quotient scales was assessed by calculating Pearson’s r linear correlation matrices. The statistical significance of the calculated r-Pearson parameter values was determined by the t-test.

The significance of Pearson’s 〖chi〗^2 statistic was used to assess the degree of correlation between the dichotomous variables and the constructed bivariate (2 × 2) tables.

The statistical significance of the differences between the mean values of the quotient variables in the two compared groups—test and control—was assessed depending on the result of the test confirming the homogeneity of variance: the parametric Student’s *t*-test for independent samples or the Cochran-Cox Z-test with independent variance estimation. 

In all statistical analyses performed, a significance level of α = 0.05 was assumed. Statistical analysis was performed using the Statistica 13.3 PL computer programme from StatSoft.

The funding source had no involvement in any part of the study. The corresponding author confirms that the authors had full access to all the data in the study and assumed final responsibility for the decision to submit it for publication.

## 3. Results

Samples from 31 patients with pre-DM and 11 healthy volunteers were evaluated.

There was no difference between the groups in the following basic parameters: age, creatinine, alanine aminotransferase (ALT), total cholesterol level (TCL), triglycerides (TG), low-density lipoprotein (LDL), high-density lipoprotein (HDL) (Table 1), sex, use of nicotine, hypertension, history of diabetes in family, using of hypertensive drugs or fibrates (Table 2).

Statistical significance was found when groups were compared for body weight, BMI, fasting glucose level (Table 1), fatty liver and use of statins (Table 2).

There was no difference in MGO concentration between the groups. The mean value of MGO was 135.44 nM (±SD = 32.67) in the pre-DM group and 143.25 nM (±SD = 17.93), *p* = 0.46 (±95% CI) in the control group (Suppl. No 1). For the group size available in the study and the MGO mean values obtained, the power of the *t*-test was β = 0.1 and the analysis of the test’s power behaviour for larger groups (size simulation) did not show any power increase. For the calculation of approximately 120 study participants, the test reaches the power (β) of around 0.12, and the power of the test does not increase despite a further increase in the number of participants.

**Figure 1:**
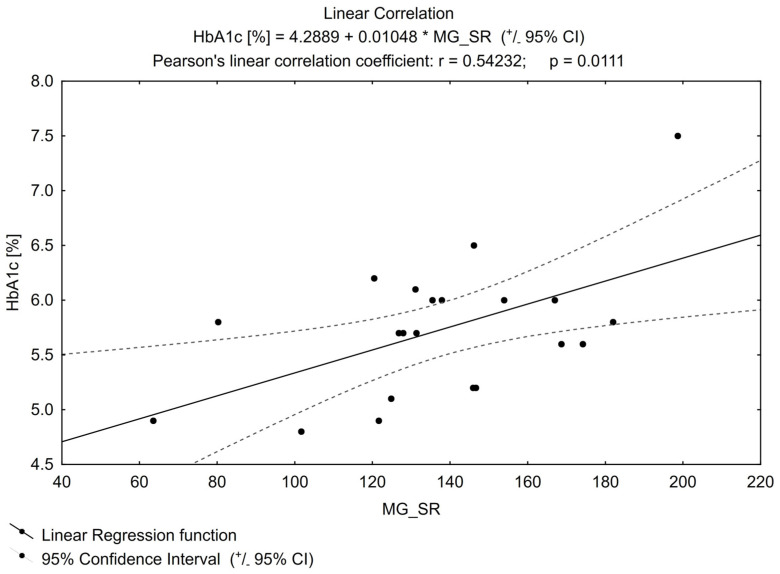
Linear correlation for HbA1c (glycated haemoglobin).

The positive linear correlation showed that the higher glycated haemoglobin (HbA1c), the higher MGO concentration (Figure 1) (*p* = 0.01). The power of the test was β = 0.74.

**Figure 2:**
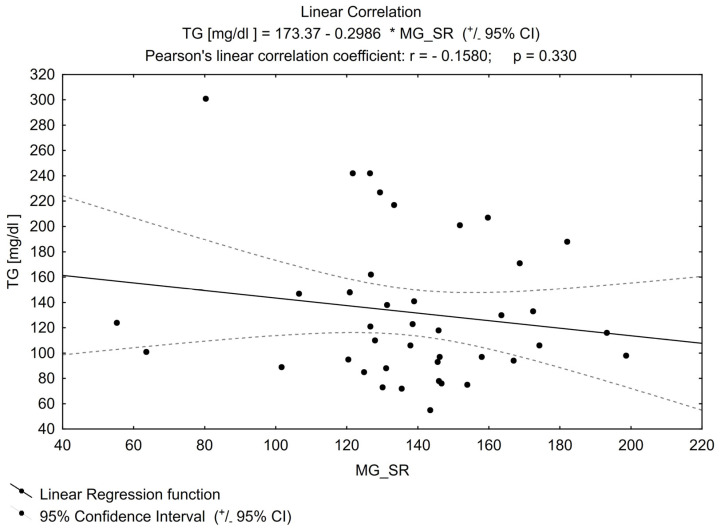
Linear correlation for TG (triglycerides).

**Figure 3:**
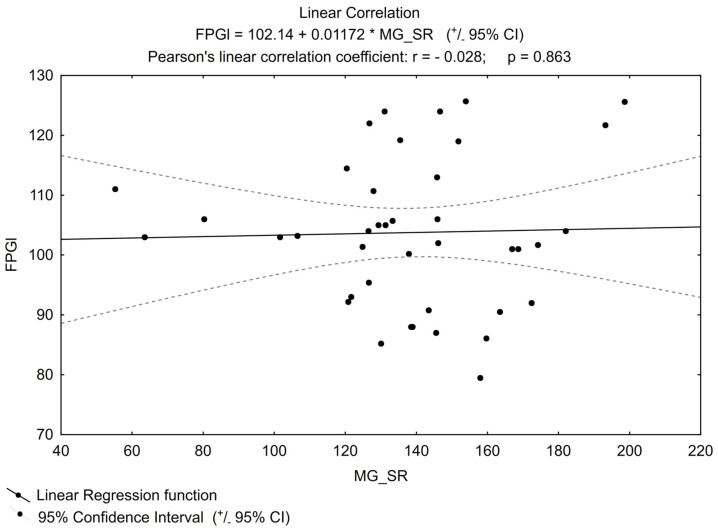
Linear correlation for FPGl (fasting plasma glucose level).

No other important correlation was found between MGO and the parameters that can impact its concentration (examples in Figure 2 and Figure 3), e.g., for TG or FPG.

## 4. Discussion

Basic and clinical data showed that the abnormal serum concentration of MGO was connected with the cardiovascular incidence in individuals with DM, which may at least partly explain a higher cardiovascular risk in this group of patients [16,23,24,25] if its contribution to the creation of AGEs, mentioned in the introduction, is considered. Higher plasma MGO levels positively correlated with the known cardiovascular risk markers like HbA1c value and albumin/creatinine ratio in the urine of patients with T2DM [26,27,28,29]. In our analysis, as in the studies mentioned above, the HbA1c value correlated positively with MGO concentration the higher the HbA1c value, the higher the MGO value. In our study, for obvious reasons, HbA1c was determined mainly in the pre-DM group (it was only incidental in the control group). According to local recommendations, glycated haemoglobin was not a parameter that allowed excluding diabetes in healthy people, but only a parameter to be used for monitoring already diagnosed disorders of carbohydrate metabolism [30]. Therefore, it was not collected for the previous study that the subjects participated in. Our study, in this regard, confirms the relationship between MGO and the aforementioned glycated blood protein for pre-DM, which was previously reported only in a group of patients with DM.

It is also emphasized in the literature that a higher concentration of MGO in plasma correlates with mortality and amputation risk in patients with critical ischemia of the lower extremities, regardless of the presence of diabetes [31]. In addition, urinary MG-H1 levels were positively correlated with cardiovascular parameters including blood pressure and lipid concentration in obese but healthy women. This has given rise to suggestions that the level of AGEs in urine, and not only the known risk factors, can serve as an independent and sensitive marker for cardiovascular risk assessment in obese individuals in the future [32]. The indication of the above correlations of MGO level with poor prognosis inspired scientists to search for new therapeutic strategies aimed at reducing the concentration of this molecule (via its detoxication or prevention of formation) which finally can help to reduce macro- and microvascular complications [33,34]. This strategy seems to have a future, because there are known substances, available on the market but used for another purpose, with MGO-binding properties, e.g., metformin (regardless of its hypoglycaemic effect) [35,36,37,38]. Several other compounds have also been found to be potentially useful in an anti-MGO strategy [39,40,41]. Therefore, there remains the question of selecting a group of individuals who will be able to benefit from such therapies, because they are characterised by an abnormal level of MGO. However, since determination of MGO concentration in serum or urine is not available in public laboratories, it may be useful to determine the phenotype of people for whom the use of MGO-lowering substances is likely to be a targeted therapy.

Taking into account the accumulation of pathological states that often accompany prediabetes and their relationship with the formation of AGEs [40], it seemed that the presence of elevated methylglyoxal levels (compared to healthy individuals) in this group was highly probable. However, the newly diagnosed, hypoglycaemic drug-naïve patients with pre-DM in our study did not have elevated MGO levels, compared to a healthy control group, although they presented features typical of the metabolic syndrome (Table 1 and Table 2): glucose levels, hepatic steatosis, body weight and BMI, which differentiated the two groups. In many countries, the aforementioned metformin, which is credited with reducing MGO levels, is used in the treatment of patients with pre-DM, although, certainly, the only intention of such recommendations is to lower glucose levels. However, in our study, the results obtained could not be a response to metformin use, because none of the participants had previously been prescribed such treatment. The fasting MGO results we found are opposite to the Hanssen N et al. study [14]. Although we are aware that sample processing can lead to an overestimation of MGO concentration, which may be released in the already collected sample during its storage or processing [20,21], it should be emphasized that the material used for our study was obtained, processed and stored under the same conditions for both groups. For this reason, we were able to compare the concentration of MGO between the study groups, and it is worth noting that the reported concentration values are within the ranges shown by other authors for the healthy population: 60–250 nM [20,22]. It should also be mentioned that, compared to our study, in the study by Hanssen N. et al., none of the analysed groups (even healthy subjects) had MGO levels within the suggested range for healthy. The other differences include the older analysed population in the Maastricht Study (almost 10 years older for each group) and statistical differences between the groups regarding the age in the Hanssen N et al. study [14]. For comparison of the MGO concentration in both groups, the analysis of the power for t-student test value in our study indicates that the power level of > 0.8 (β > 0.8) cannot be obtained regardless of sample size. This suggests no statistical significance between the groups for MGO concentration values.

An important goal of preventing chronic complications resulting from metabolic disorders is to maintain normal lipid values, with LDL being the most important point of reference. Methylglyoxal also affects this molecule, as it modifies arginine residues of the protein component of LDL, and glycation of LDL by MGO results in the formation of VLDL, the most atherogenic molecule [42,43,44]. The pre-DM group and the control group differed, as mentioned above, in terms of some characteristics typical of the metabolic syndrome that adversely affect the formation of AGEs (Table 1). In general, however, looking at the results of our work, the concentration of MGO in both groups is practically identical (even slightly lower in people with pre-DM, but without statistical significance) and comparable values of lipid metabolism parameters were shown. Looking for the cause of this surprising phenomenon and the lack of statistical significance, one can emphasize the small number of participants as the study limitation. Nevertheless, that number is typical of a preliminary study, the purpose of which is to outline the direction of further research. The only factor for which statistical significance was shown, and which could potentially “mitigate” the impact of adverse factors in the pre-DM group, was the use of statins (Table 2). This medicine, which has a known pleiotropic effect [45,46,47], was prescribed to pre-DM patients by general practitioners (not researchers) due to abnormalities in the parameters of lipid metabolism at various, unspecified times before the blood samples were collected for the study. The aim of this decision was only to correct the lipid profile (patients did not need secondary prevention) and as we found, this profile did not differentiate the groups in our study, except for the fact that healthy individuals were not treated pharmacologically with statins. This draws our attention to the potential relationship between MGO concentration and statin intake (only the pre-DM group in the study). It cannot be ruled out that such pharmacotherapy, by lowering LDL levels or independently, contributed to the regulation of AGE formation by MGO in the studied group. In the above-mentioned big study [14], in all three groups (healthy, pre-DM, and DM) lipid-lowering treatment, which could impact the results, was noted. Despite the controversies sometimes raised in the literature on this group of drugs [48] (statins inhibit cholesterol biosynthesis but at the same time they have been advocated to suppress the synthesis of the most important natural antioxidants), statins’ pleiotropic effects, including anti-inflammatory ones, and thus the influence of the lipid-lowering therapy on the concentration of MGO cannot be excluded [49].

Knowing the relationships that link hyperglycaemia and other features of the metabolic syndrome with the formation of MGO, the aim of our study was to assess whether pre-DM patients would have higher levels of this substance than healthy subjects. The obtained results, showing no differences in MGO concentration between the groups, suggest the need to analyse the effects of drugs used to correct lipid disorders on AGEs formation, as it seems impossible that people with pre-DM and healthy individuals present the same cardiovascular risk expressed by MGO value.

The limitation of our study is the number of participants which results from the nature of the study (preliminary research).

## 5. Conclusions

The higher the HbA1c, the higher the methylglyoxal value confirmed in people with pre-diabetes. Nevertheless, the concentration of methylglyoxal in studied patients with prediabetes (mean value: 135.44 nM) was within the range previously found for the healthy population (60–250 nM) by other authors [20,22]. This was also confirmed in our study when the results from individuals with pre-diabetes were compared with those from the control group. Those unexpected results could be related to the introduction of statins. This observation requires confirmation in a further study, which should be directly dedicated to this issue.

## Figures and Tables

**Table 1:** Basic characteristics of both groups (nominal variables).

Variable	pre-DM, N = 31		Control, N = 11		*p*
	Mean	±SD	Mean	±SD	
age (years)	53.65	8.35	49.91	8.37	ns
body weight (kg)	93.71	14.35	80.82	20.64	0.0284
BMI (kg/m^2^)	31.40	6.07	26.99	5.43	0.04
creatinine (mg/dL)	0.84	0.17	0.73	0.03	ns
ALT (U/L)	30.3	15.95	19.11	12.06	ns
TC (mg/dL)	197	42.64	201	27.47	ns
TG (mg/dL)	130.24	59.98	137.09	49.20	ns
HDL-C (mg/dL)	52.64	15.93	57.91	16.82	ns
LDL-C (mg/dL)	117.38	27.21	114.83	20.76	ns
FG (mg/dl)	108.85	9.44	88.81	4.44	<0.001
HbA1c (%)	5.76	0.59	5.27	0.35	ns

pre-DM—pre-diabetes; N—the number of individuals; SD—standard deviation; BMI—body mass index; ALT—alanine aminotransferase; TC—total cholesterol level; TG—triglycerides; HDL-C—high-density lipoprotein-cholesterol; LDL-C—low-density lipoprotein-cholesterol; FG—fasting glucose; HbA1c—glycated haemoglobin; ns—no statistical significance.

**Table 2:** Basic characteristics of both groups (dichotomic variables).

Variable	pre-DM, N = 31		Control, N = 11		*p*
	YES, n	YES, %	YES, n	YES, %	
Sex (woman)	12	38.71	6	54.55	ns
Nicotinism	7	22.58	2	18.18	ns
Hypertension	17	54.84	3	27.27	ns
Fatty liver	18	64.29	1	9.09	0.001
Family history for DM	12	38.71	3	27.27	ns
ACEI/ARB	10	33.33	3	27.27	ns
Beta-blockers	8	26.67	2	18.18	ns
Calcium-blockers	3	10	0	0	ns
Diuretics	4	13.33	0	0	ns
Statins	10	32.26	0	0	0.03
Fibrates	1	3.23	0	0	ns

pre-DM—pre-diabetes; N—the number of individuals; n—number, %—percent of individuals meeting the criterion; DM—diabetes mellitus; ACEI—Angiotensin-converting enzyme inhibitors; ARB—Angiotensin receptor blockers; ns—no statistical significance.

## Data Availability

Basic data and statistical analysis are available as a Supplementary Material: Figure S1: Basic data.

## Supplementary Materials

The following supporting information can be downloaded at https://www.mdpi.com/article/10.3390/biomedicines11112968/s1. Figure S1: Basic data.
