# Supplementary figures and images for: Analysis of Methylglyoxal Concentration in a Group of Patients with Newly Diagnosed Prediabetes
Source: Biomedicines. 2023 Nov 3;11(11):2968. doi: 10.3390/biomedicines11112968 (PMC10669086; doi:10.3390/biomedicines11112968)

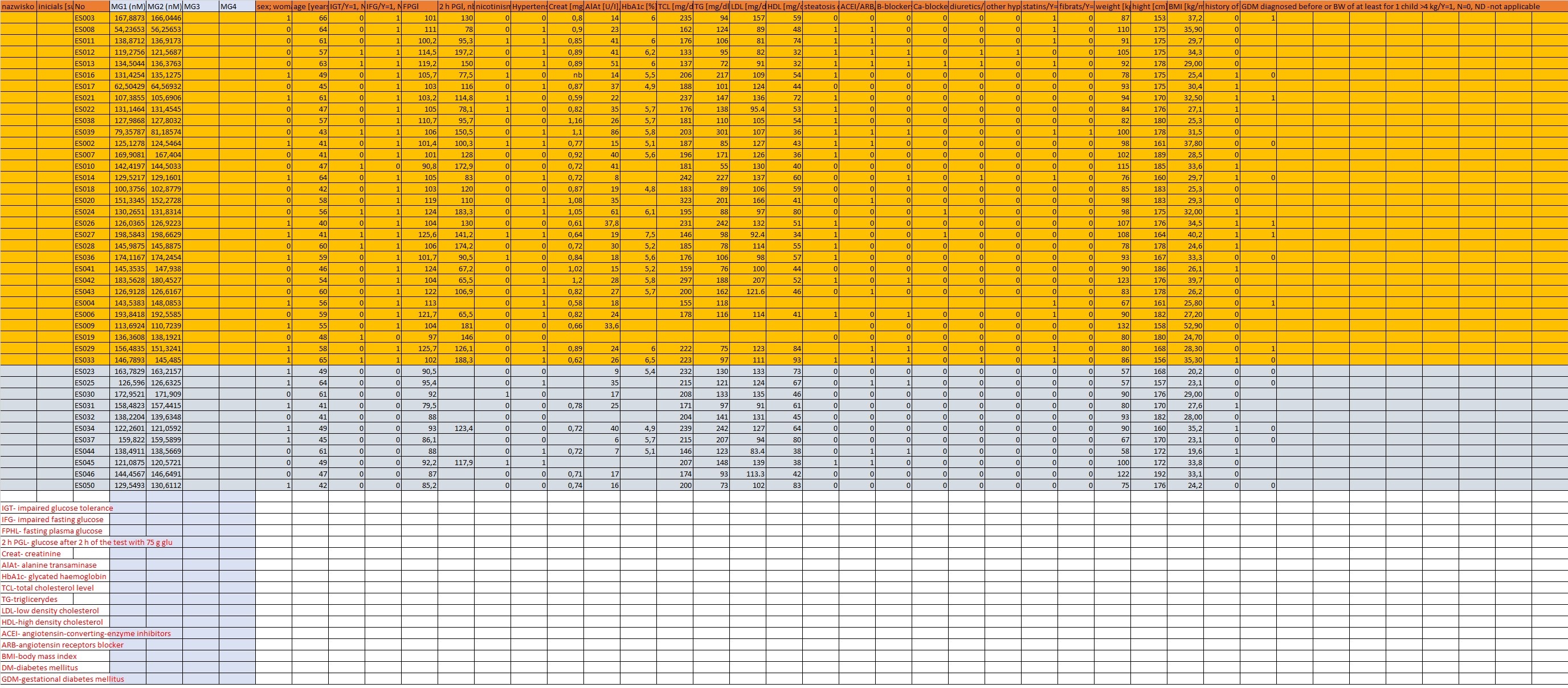

Supplement: Supplementary file 1 [file biomedicines-11-02968-s001.zip › biomedicines-2699184-supplementary.jpg]
